# Simple kinetic method for assessing catalase activity in biological samples

**DOI:** 10.1016/j.mex.2021.101434

**Published:** 2021-07-01

**Authors:** Ali A. Farman, Mahmoud Hussein Hadwan

**Affiliations:** Chemistry Dept., College of Science, University of Babylon, Hilla city, Babylon Governorate, p.o. 51002, Iraq

**Keywords:** Biological fluids, Catalase activity, Hydrogen peroxide, Pyrogallol red, Molybdenum

## Abstract

A novel kinetic method for measuring catalase activity in biological samples was evaluated. The principle of the current method is based on the oxidation effect of unreacted hydrogen peroxide (H_2_O_2_) on pyrogallol red (PGR) using the catalytic effects of molybdenum. The decrease in the absorbance of PGR in the presence of H_2_O_2_ with time from 0.5 to 4.5 min was directly proportional to the concentration of H_2_O_2_, and, in turn, directly proportional to catalase activity. Erythrocyte lysate homogenates were used to measure catalase activity and the results of the current method were significantly correlated to those of the ammonium peroxovanadate method. The 3.1% within run and 4.7% between run coefficients of variation indicated the high precision of the present novel method. The validation process confirmed that the diagnostic method is appropriate for different types of biological samples. Here, we describe a rapid, relatively easy, and reliable method for measuring catalase activity. The assay could be applied as a diagnostic tool and is suitable in research contexts.

• A novel kinetic method for measuring catalase activity in biological samples was evaluated.
• The validation process confirmed that the diagnostic method is appropriate for different types of biological samples.
• The assay could be applied as a diagnostic tool and is suitable in research contexts.

## Graphical abstract

**Figure:**
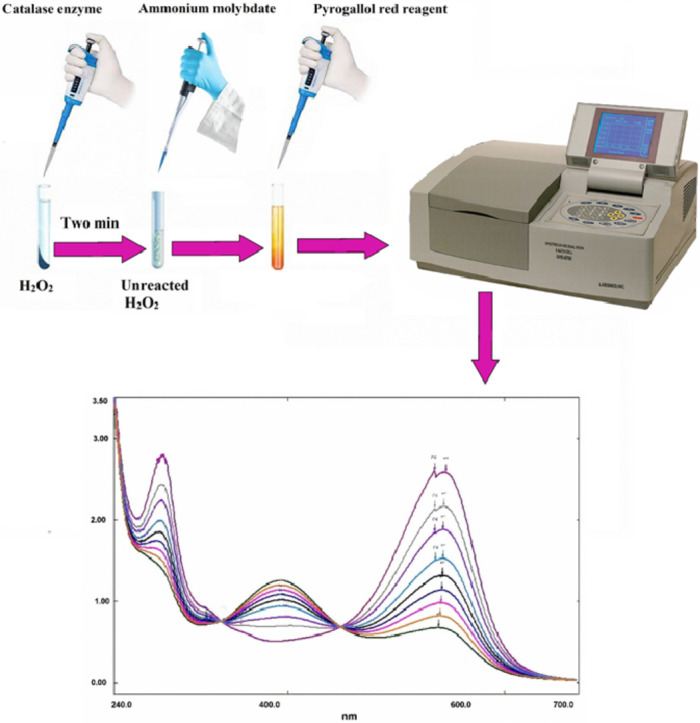

**Specifications Table**

**Table:** 

**Subject Area**	Biochemistry, Genetics and Molecular Biology
**More specific subject area**	*Enzymology*
**Method name**	**Assessing catalase activity in biological samples**
**Name and reference of original method**	*N/A*
**Resource availability**	Included in each section of the method

**Method details**

Fig. 1 elucidates the steps of the current method.

**Fig. 1:**
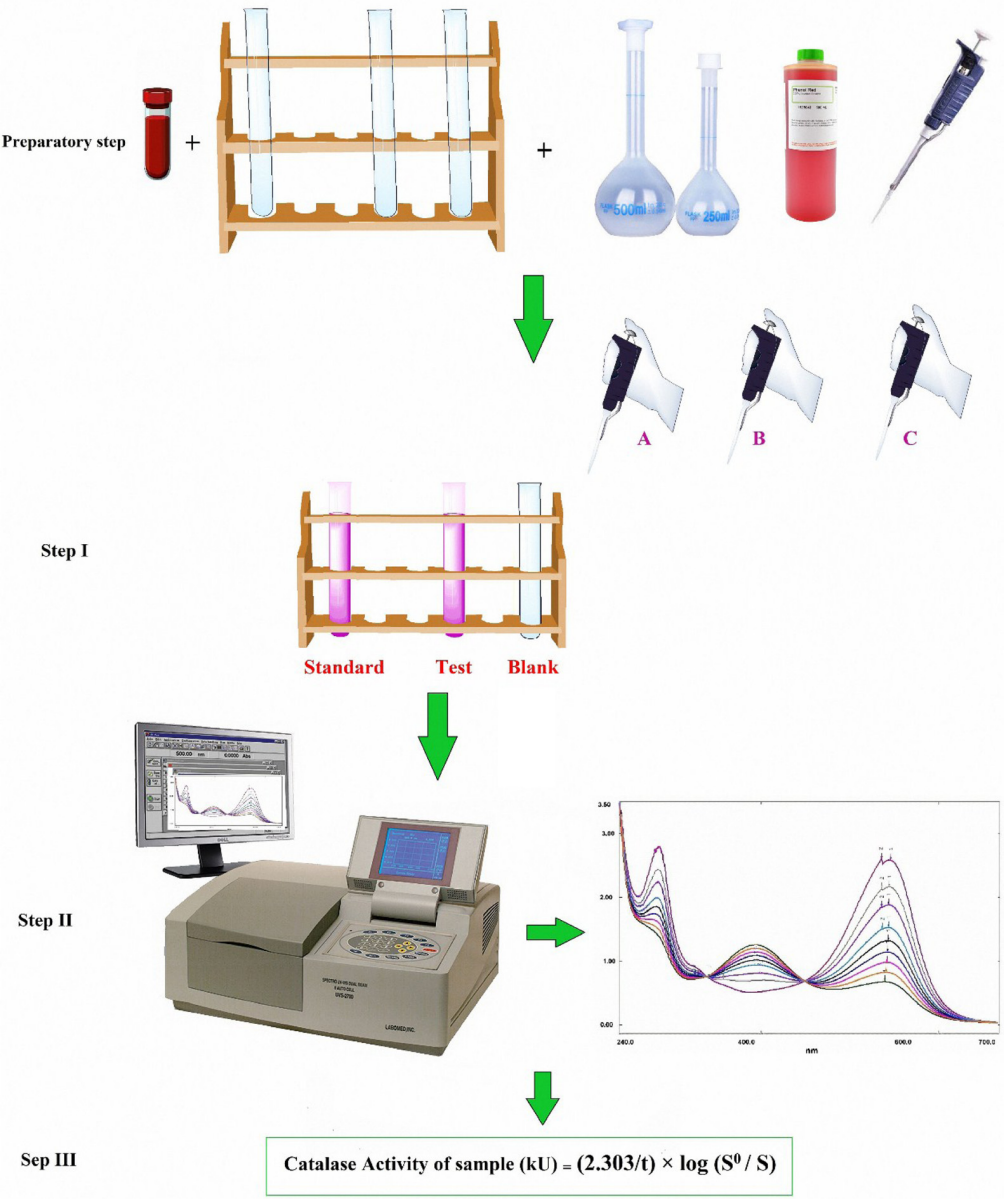
Schematic showing the details of the current colorimetric kinetic method that used to assay catalase enzyme activity. Preparatory step includes preparation samples, equipment and reagents. Step I explains the sequence of addition reagents; A: addition substrate (2000 µl of hydrogen peroxide), B: addition 1000 µl of catalase source enzyme. Thereafter, test tubes were vortexed and incubated at 37 °C for two min. At the end of incubation time, 500 µl aliquots of each tube were withdrawn to another clean test tube containing 300 µl of ammonium molybdate, C: addition pyrogallol red reagent. Step II explains the monitor of absorbance as a function to the time. The zero time of the assessment reaction was recorded as the moment at which the total quantity of pyrogallol red solution had been added to the enzymatic reaction. The absorbance decrement as a function of time ∆As was recorded against a distilled water as a blank for 0.54.5 min from start of the assessment reaction between PGR and un-reacted hydrogen peroxide. Step III explains the application of first order reaction equation to obtain catalase enzyme activity.

## Materials and methods

### Chemicals

All chemicals and biochemical reagents were of analytical grade and were purchased from standard chemical commercial providers. The standard catalase was purchased from HiMedia Laboratories (product code TC037; New Delhi, India).

### Reagents

PGR reagent (1 mM): 0.04 gm of the reagent was dissolved in 50 ml distilled water and 50 ml ethanol. The stoke solution was then diluted to obtain the target concentration: 0.05 mM.

Phosphate buffer solution (PBS), fresh H_2_O_2_, catalase standard solution, erythrocyte lysates, and tissues were prepared according the methods described by Hadwan and Ali [1]. 0.170 ml of 30% H_2_O_2_ was dissolved in 100 ml PBS and the final concentration was calibrated to 15-mM at 240 nm based on the molar extinction coefficient of H_2_O_2_ (43.6 *M* ^−^ ^1^ cm^−1^). Ammonium molybdate (2.5 mM)**:** 50 mg of (NH_4_)_2_MoO_4_ was dissolved in 100 ml of distilled water.

The standard catalase solution consisted of 20 mg of standard catalase dissolved in 100 ml of PBS (pH 7.0; 50 mM). Catalase activity was adjusted to 3 U mL^−1^ using the peroxovanadate method as described by Hadwan and Ali [1].

### Blood samples

Three milliliters of whole blood were transferred to a heparinized tube and used to prepare the erythrocyte lysates. After centrifugation at 400 × *g* for 10 min, buffy coat cells and plasma were discarded. Five-hundred microliters of 0.9% sodium chloride solution was used to wash the RBCs obtained three times. Subsequently, 2 ml of ice-cold double distilled water was mixed with 500 µl of the erythrocyte mixtures. The obtained mixture was vortexed for 10 s and stored at 4 °C for 15 min in the dark. Finally, the obtained stock hemolysate was resuspended in 50 mM PBS and diluted with a dilution factor of 500. The diluted hemolysate solutions were used as the source of catalase activity.

### Tissue preparation

Male albino rats and mice were obtained from the laboratory of animal house, Bioscience department, Babylon University. Broiler chicken were purchased from a local market. Before the measurement of catalase activity in tissues, animal liver tissues were surgically enucleated. NaCl solution (0.9%) (w/v) was used to wash blood and other contaminants immediately from the liver, which were then homogenized using cold 1.15% (w/v) KCl in a glass homogenizer. Afterward, homogenate solutions were filtered and diluted (at a ratio of 1:500) with 50 mM PBS.

## Ethical committee

Iraq: Ethics Committee (University of Babylon/ College of Science), Reference number of approval: 6335 Date: 12/9/ 2018.

### Instruments

The present study used a Shimadzu 1800 spectrophotometer (Shimadzu Scientific Instruments, Columbia, MD, USA) for spectrophotometric analyses.

### Detailed of procedure

Table 1

1 Two ml of hydrogen peroxide was added to test tube and standard tube.
2 Subsequently, one ml of sample containing catalase enzyme or distilled water were added to test tube or standard tube, respectively.
3 Test tubes were vortexed and incubated at 37 °C for two min.
4 Thereafter, 500µl aliquots of each tube were withdrawn to another clean test tube containing 300µl of ammonium molybdate.
5 The test tubes were mixed well, and then 3 ml pyrogallol red were added to each tube.
6 The zero time of the assessment reaction was recorded as the moment at which the total quantity of pyrogallol red solution had been added to the test tube.
7 The next step includes transfer a suitable quantity of the reaction solution into the cuvette within 30 s. The absorbance decrement as a function of time ∆As was recorded against a distilled water as a blank for 0.5–4.5 min from start of the assessment reaction between PGR and un-reacted hydrogen peroxide.

**Table 1:** The steps of the procedure that used for measuring the catalase enzyme activity.

Reagents	Test	Standard
Hydrogen peroxide	2000 µl	2000 µl
Catalase source sample	1000 µl	——
Distilled water	——	1000 µl

Test tubes were vortexed and incubated at 37 °C for two min. At the end of incubation time, 500 µl aliquots of each tube were withdrawn to another clean test tube containing 300 µl of ammonium molybdate. The test tubes were mixed well, and then add:		

Pyrogallol Red reagent	3000 µl	3000 µl

The zero time of the assessment reaction was recorded as the moment at which the total quantity of pyrogallol red solution had been added to the enzymatic reaction. The next step includes transfer a suitable quantity of the reaction solution into the cuvette within 30 s. The absorbance decrement as a function of time ∆As was recorded against a distilled water as a blank for 0.5–4.5 min from start of the assessment reaction between PGR and un-reacted hydrogen peroxide.		

The protocol was summarized in Table 1.

### Calculation

Catalase activity was determined based on the rate constant of a first-order reaction (k) equation:

(1) $$\text{CatalaseActivity}\;\text{of}\;\text{test}\;\text{kU}=\frac{2.303}{t}*\log\frac{S^{\circ }}{S}$$

t: time

S°: ∆A of standard tube

S: ∆A of test tube

∆*A* = (Absorbance at *t* = 0.5 min–Absorbance at *t* = 4.5 min)

## Method validation

**Fig. 2:**
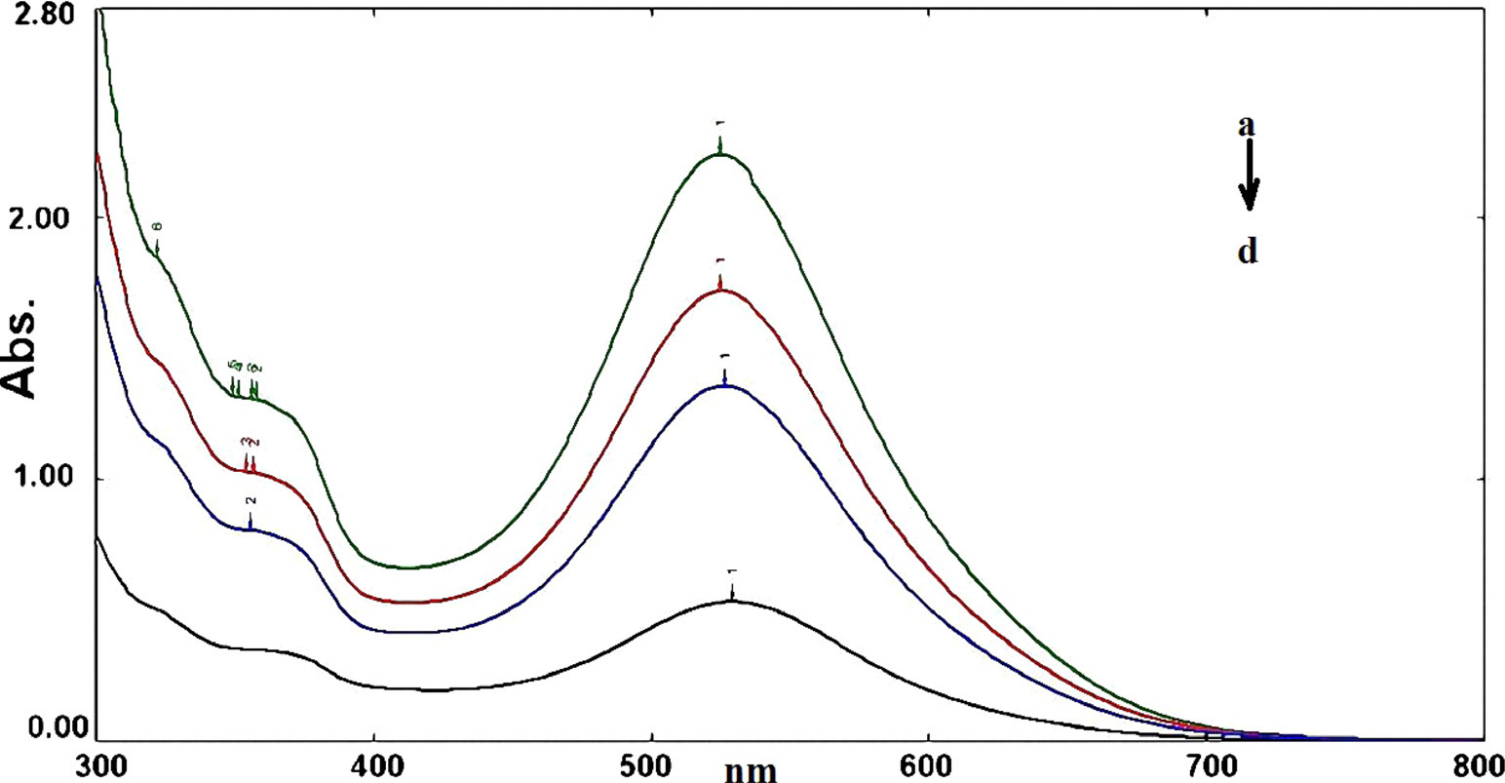
Absorption spectra of the different concentrations of PGR, (a) 0.1 mM PGR (b) 0.08 mM PGR (c) 0.05 mM PGR (d) 0.02 mM PGR.

**Fig. 3:**
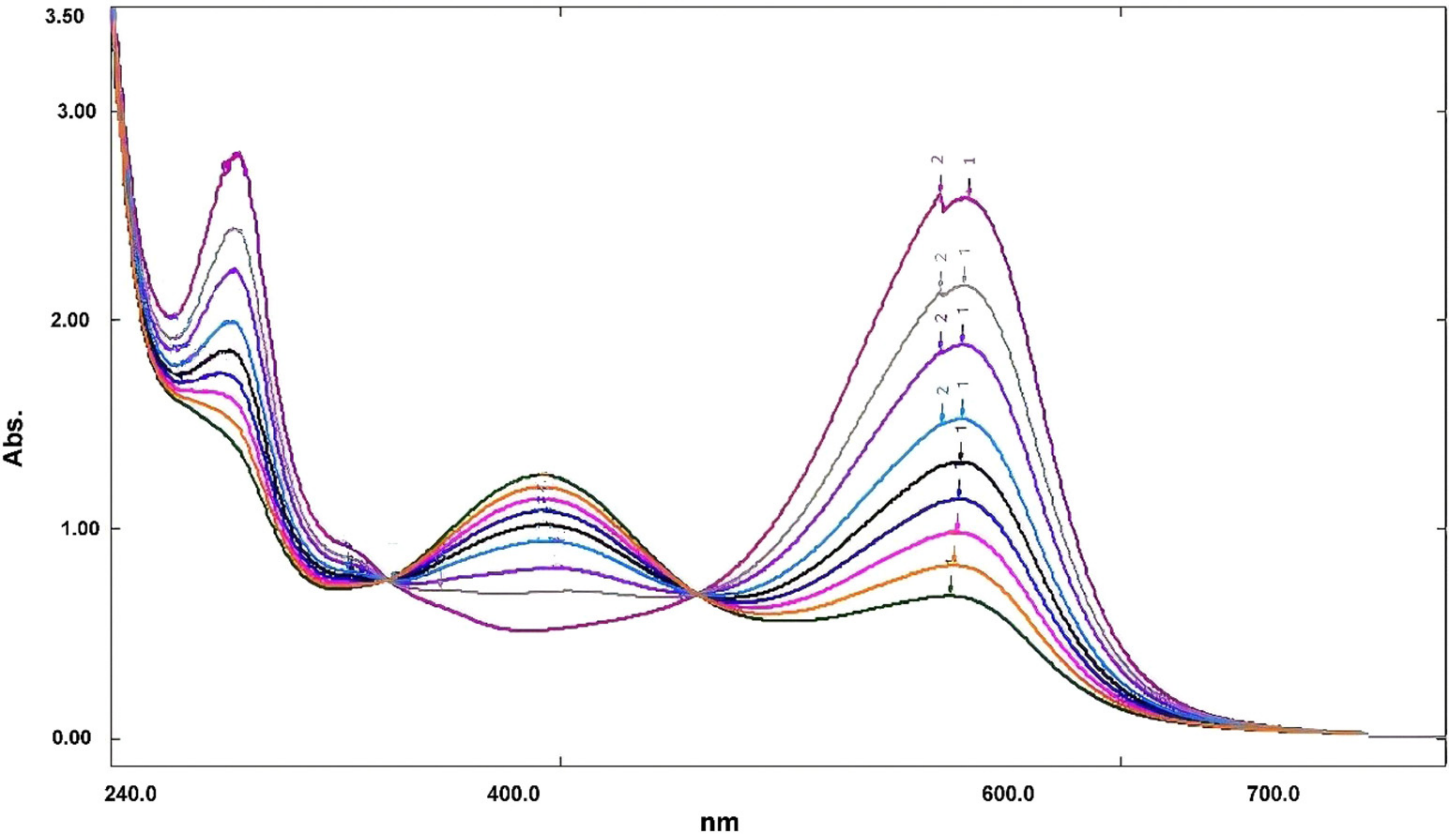
Variation of the PGR-H_2_O_2_-Mo(VI) system as a function to the time. Conditions of reaction: PGR concentration 1 mmol/L, pH 7.0, H_2_O_2_ concentration 0.020 mol/L, 2.5 mM Mo(VI) at 30 °C, time interval for each scan 70 s.

Ammonium molybdate reagent was used to halt the catalase reaction. Ammonium molybdate reacts with H_2_O_2_ to form singlet oxygen (^1^O_2_) [2]. Subsequently, ^1^O_2_ molecules react with reduced PGR to form the oxidized form; as shown in Eqs. (1), (2), and (3). H_2_O_2_ dissociation is directly proportional to catalase activity and a decrease in absorbance of the characteristic PGR band (545 nm) at pH 7.0 was used to monitor the rate of PGR oxidation as shown in Figs. 2 and 3.

(1) MoO_4_^2−^ + nH_2_O_2_ ⇆ MoO_4-n_(O_2_)_n_^2−^+nH_2_O

(2) $${\text{MoO}}_{\text{4-n}}{\left(O_{2}\right)}_{n}^{2-}\overset{\text{n=2-4}}{⟶}{}^{1}O_{2}+{\text{MoO}}_{\text{6-n}}{\left(O_{2}\right)}_{\text{n-2}}^{2-}$$

(3) $${}^{1}O_{2}+\text{Reduced}\;\text{PGR}\to \text{Oxidized}\;\text{PGR}$$

The catalytic effect of Mo on the oxidation state of PGR with H_2_O_2_ has been used previously to determine the concentration of Mo [3].

## Selectivity

To investigate the potential biochemical interference that could cause variations in catalase activity, interference was evaluated according to the method of Hadwan and Abed [4]. The investigation of potential sources of interference for catalase activity tested 10 substances consisting of 1 ml of catalase with known activity (30 U/ml) and 9 ml of the potential substance dissolved in 50 mM PBS (pH 7.4). The peroxovanadate method, as demonstrated by Hadwan and Ali [1], was used to calibrate the observed catalase activity. Final enzyme activity was 3 U mL^−1^. Table 2 lists the effects of different types of interference on the catalase activity.

**Table 2:** Effects of several probable interferences on assessment of the catalase activity using the current new method (PGR/Mo method).

Chemicals	Concentration of chemical	Added catalase Unit	Found catalase Unit	Relative error (%)
Glucose	120 mg dl^−1^	3	3.01	0.33
Fructose	120 mg dl^−1^	3	3.05	1.6
Cellulose	120 mg dl^−1^	3	3.01	0.33
Methionine	50 µM	3	3.01	0.33
Valine	50 µM	3	2.9	−3.33
Heparin	78.4 USP/10 mL	3	3	0.0
Threonine	50 µM	3	2.9	−3.33
Ascorbic acid	50 µM	3	3.1	3.33
Uric Acid	50 µM	3	3.05	1.6
EDTA	20.0 µM	3	3	0.0

## Precision

The assessment of catalase activity in homogenized diluted RBC solutions was used to evaluate the reliability of the PGR method (PGR/Mo method). Catalase activity was assessed using the current method and then compared with values obtained using the peroxovanadate method as described by Hadwan and Ali [1]. Similar buffers, reagents, and samples were used in both methods. The PGR/Mo method demonstrated good reliability as shown in Table 3.

**Table 3:** Reliability of the pyrogallol red assay (PGR/Mo method).

	*n.*	Mean (±SD): U.mL^−1^	95% Confidence Interval	CV%
Within-run	20	3.2 ± 0.1	3.2 ± 0.088	3.1%
Between-run	20	3.15 ± 0.15	3.15 ± 0.1446	4.7%

## Accuracy

In addition, the data obtained from the current method were significantly correlated with those from the peroxovanadate assay as shown in Table 4.

**Table 4:** The statistical correlation between the catalase activity assessment that obtained by applied the PGR/Mo method and the peroxovanadium method.

The numbers of measurements	20
Mean of catalase activity that assessed by the present method U.mL^−1^.	2.8
Mean of catalase activity that assessed by the peroxovanadate method U.mL^−1^.	2.92
Mean of catalase activity that assessed by both methods U.mL^−1^.	2.86
The regression coefficient B	0.9863
The regression coefficient A	−0.0552
The correlation coefficient	0.9986

## Lower limit of quantification and linearity

PBS (0.05 mM, pH 7) was used to prepare catalase for activity recovery measurements. Catalase activity has been standardized previously using the peroxovanadate method [1]. When the catalase solution was added to the reaction with activity ranging from 0.1 to 5.0 U mL^−1^, the recovery rate of catalase activity was more than 95%, while the recovery rate decreased to 88.4% in at 6 U mL^−1^ activity (Table 5).

**Table 5:** The recovery rates of catalase in different solutions with varying enzyme activity.

Contents of catalase enzyme	Catalase enzyme activity added U mL^−1^	Catalase enzyme calculated activity U mL^−1^	Catalase enzyme observed activity a U mL^−1^	Recovery%
Enzymatic sample	—–	—–	0.5	—–
Catalase enzyme added + enzymatic sample	0.1	0.6	0.58	96.7%
Catalase enzyme added + enzymatic sample	1.0	1.5	1.55	96.7%
Catalase enzyme added + enzymatic sample	1.5	2.0	1.95	97.5%
Catalase enzyme added + enzymatic sample	2.5	3.0	2.9	96.7%
Catalase enzyme added + enzymatic sample	3.5	4.0	4.05	101.25%
Catalase enzyme added + enzymatic sample	4.5	5.0	4.85	97.00%
Catalase enzyme added + enzymatic sample	5.5	6.0	5.3	88.4%

a mean of triplicate determinations.

The findings presented in Table 5 suggest that the linearity of the (PGR/Mo method) assay is approximately 4.85 U mL^−1^. Limit of detection (LOD) was equaled to 0.012 U mL^−1^, while, the limit of quantification (LOQ) was equaled to 0.04 U mL^−1^. depending upon these findings, the linearity, LOQ and LOD for the present assay were better than those of other published assays [1], [2], [3], [4], [5], [6], [7], [8]. On the other hand, the accuracy, within-run precision and between-run precision were compatible. In expressions of the analytical methodology, the current protocol is desirable to measure catalase enzyme activity compared with other analytical protocols.

In the presence of catalase, the H_2_O_2_ was converted to H_2_O and O_2_, which were indicated by the decreased absorbance values at the distinguishing PGR band (545 nm) at pH 7.0; (Fig. 3). The optimal incubation time for assessing catalase activity and the optimal concentrations of PGR and Mo were assessed by determining the activity in a 3 U mL^−1^ solution (Product code: TC037, HiMedia, New Delhi, India) using the current method. PBS was used (0.05 mM, pH 7) to prepare catalase for the experiments. In addition, the catalase activity was standardized using the peroxovanadate method as demonstrated by Hadwan and Ali [1]. Data in Table 6 show that the appropriate incubation time was 120 s, data in Table 7 show the optimal PGR concentration, while data in Table 8 show the optimal Mo concentration.

**Table 6:** The correlation between incubation time and catalase activity.

Prepared catalase enzyme activity	3	3	3	3	3	3
Incubation time (*sec*)	60	120 b	180	240	300	360
Obtained catalase enzyme activity a	1.8 ± 0.6	3.0 ± 0.1	3.0 ± 0.3	2.7 ± 0.5	2.2 ± 0.6	2.0 ± 0.3

a mean of triplicate determinations.

b optimal incubation time.

**Table 7:** The correlation between PGR concentration and catalase activity.

Prepared catalase enzyme activity	3	3	3	3
PGR concentration	0.15 mM,	0.1mM	0.05 mM b	0.03mM
Obtained catalase enzyme activity a	1.8 ± 0.6	2.7 ± 0.4	3.0 ± 0.1	2.7 ± 0.5

a mean of triplicate determinations.

b optimal PGR concentration.

**Table 8:** The correlation between ammonium molybdate concentration and catalase activity.

Prepared catalase enzyme activity	3	3	3	3	3
Ammonium molybdate concentration	1.0 mM,	1.5mM	2.0mM	2.5 mM b	3.0mM
Obtained catalase enzyme activity a	2.8 ± 0.6	2.7 ± 0.4	3.0 ± 0.3	3.0 ± 0.1	2.7 ± 0.5

a mean of triplicate determinations.

b optimal ammonium molybdate concentration.

## Dilution integrity and calibration curve

**Fig. 4:**
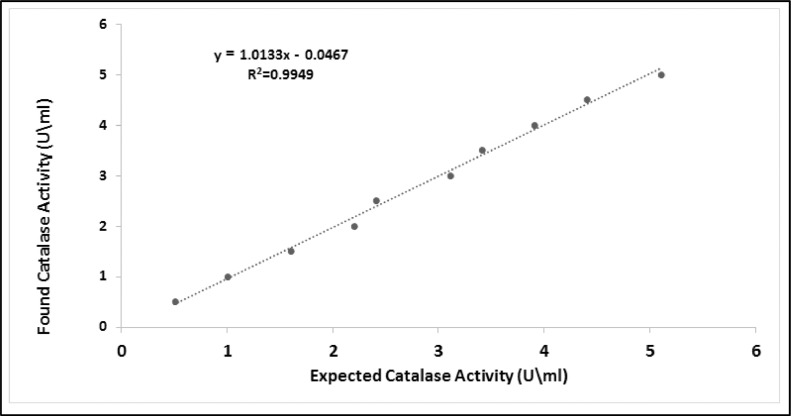
The comparison between catalase enzyme activity of RBC homogenates that assessed by utilize the PGR/Mo method and peroxovanadate method.

Numbers of dilutions of homogenate RBC were used to calculate the sensitivity of the current method. Fig. 4 illustrates a comparison between expected and measured catalase activity. The expected enzyme activity was calculated using the current method while the actual enzyme activity was calculated using the peroxovanadate method. The expected activity in the presence of RBC homogenates were linearly correlated (*r* = 0.9949) with the measured catalase activity. In addition, the obtained linear curve passed through the origin.

## Matrix effect

**Fig. 5:**
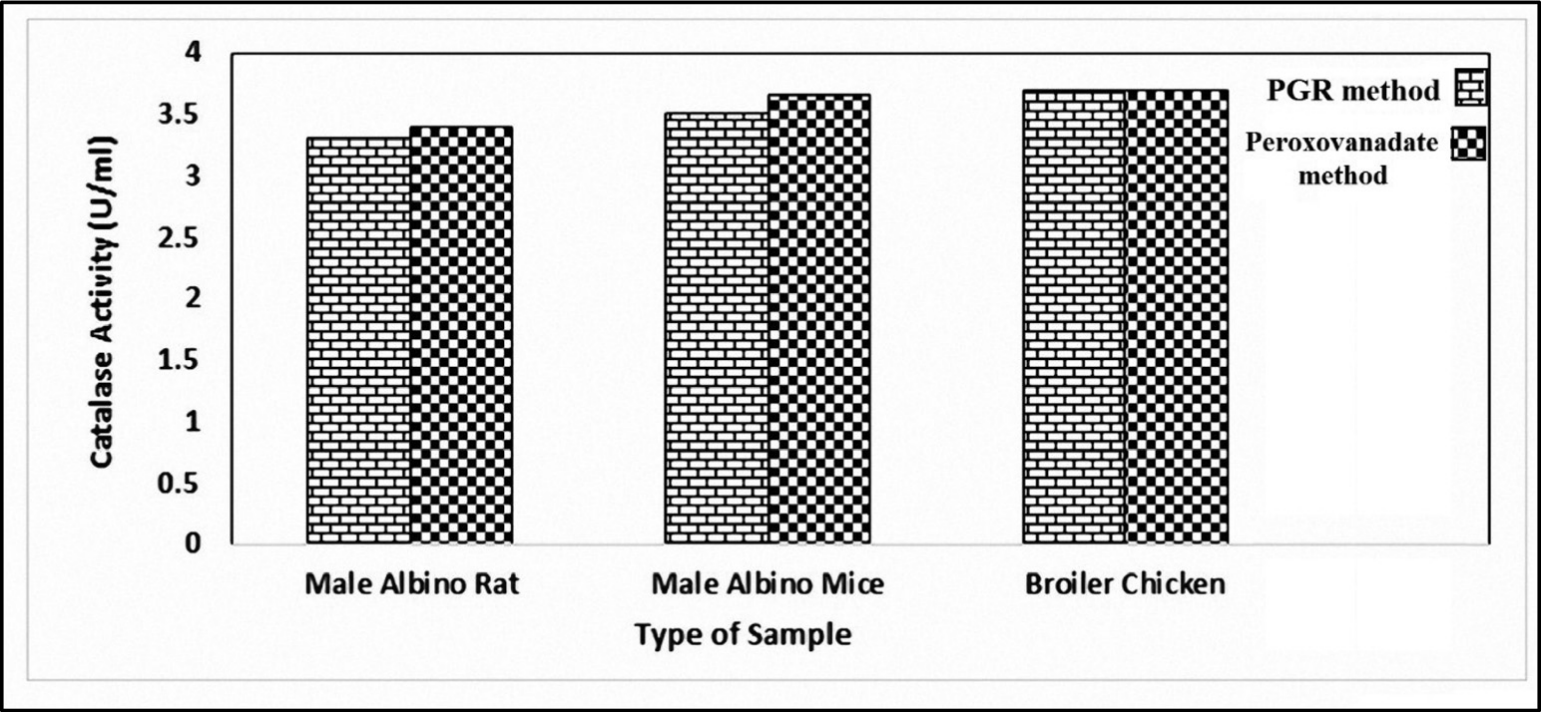
The comparison of PGR and peroxovanadate methods for assessment of catalase activities in (1-500) dilution of homogenate tissues.

The PGR/Mo assay was applied for the measurement of catalase activity of homogenous liver tissue from male albino rats, male albino mice, and broiler chicken. As expected, liver tissue exhibited high catalase activity (Fig. 5). Catalase activity is a good tool for assessing liver function and resistance to oxidative stress [5]. In addition, numerous studies have reported that catalase activity in the livers of albino mice, albino rats, and broiler chickens could be an index for liver function [6,7].

Liver catalase activity has been used to estimate oxidative stress levels in broiler chicken [8,9]. In addition, Kikusato and Toyomizu [10] used catalase measurements in chicken liver to assess the different effects of heat stress on oxidative status of skeletal muscle with different muscle fiber compositions in broiler chicken while Zoidis et al., [11] used catalase activity in the livers of broiler chicken to investigate the effects of cadmium and selenium on the gene expression of liver antioxidant proteins and the composition of breast muscle fatty-acids. Overall, a comparison between the reliability of the PGR/Mo method and the peroxovanadate method (Fig. 5) for the assessment of catalase activity in tissue homogenates showed compatibility between the two methods.

The present (PGR/Mo) assay has numerous advantages over former assays for catalase activity assessment in biological tissues. First, the current method is free from the negative factors associated with the uv-spectrophotometric method. The PGR/Mo method used H_2_O_2_ with a concentration equivalent to 15 mM while the uv-spectrophotometric method used H_2_O_2_ with a concentration equivalent to 70 mM. High concentrations of H_2_O_2_ alter catalase active site structure, which inhibit catalase activity instantly [12]. In addition, the PGR/Mo method is free from interference that arises from the presence of proteins, sugars, or DNA that absorb UV light because it relies on a decrease in absorbance of the distinguishing PGR band at 545 nm to assess catalase activity.

Abderrahim et al., [13] applied PGR as an optical probe for the detection of unreacted H_2_O_2_ based on HRP-catalyzed oxidation to develop a sensitive PGR-based catalase activity assay. The present PGR/Mo method has numerous advantages over the Abderrahim et al. method (PGR/HRP method). First, the PGR/Mo method uses ammonium molybdate to react with H_2_O_2_ to form a complex compound and halt the enzymatic reaction completely; however, in the PGR/HRP method, there is competition between catalase and HRP. The catalase will certainly be dominant because it has a much greater turnover number than HRP. Catalase has the highest turnover number value of 40,000,000 (per second per molecule of enzyme) [14] while HRP has a turnover number value of 25,000 (per second per molecule of enzyme) [15], i.e., catalase has a turnover number approximately 1600-fold that of HRP. Secondly, the incubation time of the PGR/HPR method is 30 min, while it is 2 min in the PGR/Mo method. In addition, compared with numerous previously established procedures, the current PGR/Mo method is relatively inexpensive, could be made available as assay kits, and does not require elaborate procedures to use.

Our assessments of catalase activity using the simple method demonstrated high accuracy and precision even using high concentrations and following interference with various chemicals, in addition to low H_2_O_2_ concentrations. Based on the data obtained, the PGR/Mo assay facilitates the assessment of catalase activity at low substrate concentrations. In addition, it is a sensitive method for the determination of the H_2_O_2_ concentrations.

Performance of the current method was achieved according to Guideline on bioanalytical method validation that described by Committee for Medicinal Products for Human Use [23]. The results of application the guideline on bioanalytical method validation were elucidated in Table 9.

**Table 9:** Performance output of the current method.

*n*	Parameter	Output
1	Selectivity	The method is selective according to the results of Table 2.
2	Precision	The precision of the method was proved by the results of Table 3.
3	Accuracy	The accuracy of the method was verified by the results of Table 4.
4	Lower limit of quantification	Low limit of quantification (LOQ) was equaled to 0.04 U mL^−1^.
5	Dilution integrity	The dilution integrity of the method was elucidated in Fig. 3.
6	Linearity	The linearity of the method was ranging from 0.1 to 5.0 U mL^−1^
7	Calibration curve	The calibration curve of catalase enzyme was shown in Fig. 3.
8	Matrix effect	The matrix effect was studied by assay catalase activity of homogenous liver tissue from male albino rats, male albino mice, and broiler chicken. The method is free from matrix effect.

## General background information

Catalase (hydroperoxidase EC 1.11.1.6) is an antioxidant enzyme that protects cells from the toxic effects of hydrogen peroxide (H_2_O_2_) by decreasing the concentrations of free radicals and oxygen species [16]. The structure of catalase consists of four identical subunits each containing a single ferriprotoporphoryn group. In the first line of enzymatic antioxidant protection in peroxisomes, while catalase plays the major role via the conversion of H_2_O_2_ into molecular oxygen and water [17]. Catalase is found in human tissues, particularly in blood, liver, and kidney [18], and was one the first enzymes to be isolated and purified [19].

There are four types of methods of assessing catalase activity. The first type is based on spectrophotometric techniques, which monitor changes in concentrations of H_2_O_2_ in solutions (always more than 30 mM) at 240 nm. There are two limitations of applying the uv-spectrophotometric method [20]. High concentrations of H_2_O_2_ alter the structures of catalase active sites, which could inhibit catalase enzyme activity. In addition, proteins and DNA absorb UV light; therefore, the uv-spectrophotometric method is unsuitable for measuring catalase activity in biological tissues [12].

The second type of methods includes complex methods such as the chemiluminescent method, oxygen electrodes, low-flow gas meters, potentionmetry, iodometry, titrimetry, and polarimetry. Such procedures have numerous drawbacks including the need for expensive instruments, low limits of detection, and prolonged analysis times.

The third type of methods apply different probes in the presence of horseradish peroxidase (HRP) to detect residual H_2_O_2_ concentration [13]. A kinetic and competitive reaction between catalase and HRP for H_2_O_2_ has been applied as a probe for H_2_O_2_ [21]. In other methods, a highly specific and sensitive Amplex Red (N-acetyl-3,7-dihydroxyphenoxazine) has been used in a non-competitive fluorimetric/UV–Vis reaction to measure catalase activity via the quantification of the un-reacting H_2_O_2_ [22].

Spectrophotometric methods represent the fourth type of catalase assessment methods. Spectrophotometric methods use diverse reagents to form colored complexes that absorb light at the visible spectrum such as the carbonato cobaltate(III) ([C*o*(CO_3_)_3_]Co) complex that absorbs light at 440 nm [16] and the peroxovanadate complex (NH_4_[VO(O_2_)SO_4_) that absorbs light at 452 nm [4].

The present paper illustrates a simple kinetic method for determining catalase activity based on the measurement of H_2_O_2_ spectrophotometrically using Pyrogallol Red (PGR) as a sensitive prop and the catalytic effects of molybdenum (Mo). The method is free from interference, can be easily applied in research contexts, and suitable as a clinical analytical tool (Fig. 5).

## Declaration of Competing Interests

The authors declare that there are no conflicts of interest
